# Towards an integrated account of the development of self-regulation from a neurocognitive perspective: A framework for current and future longitudinal multi-modal investigations

**DOI:** 10.1016/j.dcn.2020.100829

**Published:** 2020-07-25

**Authors:** Matthijs Vink, Thomas Edward Gladwin, Sanne Geeraets, Pascal Pas, Dienke Bos, Marissa Hofstee, Sarah Durston, Wilma Vollebergh

**Affiliations:** a Experimental Psychology, Helmholtz Institute, Utrecht University, Utrecht, the Netherlands; b Developmental Psychology, Utrecht University, Utrecht, the Netherlands; c UMC Utrecht Brain Center, University Medical Centre Utrecht, Utrecht University, Utrecht, the Netherlands; d Behavioural Science Institute, Radboud University Nijmegen, Nijmegen, the Netherlands; e Institute for Lifecourse Development, University of Greenwich, London, UK; f Department of Child and Adolescent Studies, Utrecht University, Utrecht, the Netherlands; g Department of Interdisciplinary Social Science, Utrecht University, Utrecht, the Netherlands

**Keywords:** Self-regulation, Development, Effortful control, Executive functions, Early intervention

## Abstract

Self-regulation is the ability to monitor and modulate emotions, behaviour, and cognition in order to adapt to changing circumstances. Developing adequate self-regulation is associated with better social coping and higher educational achievement later in life; poor self-regulation has been linked to a variety of detrimental developmental outcomes. Here, we focus on the development of neurocognitive processes essential for self-regulation. We outline a conceptual framework emphasizing that this is inherently an integrated, dynamic process involving interactions between brain maturation, child characteristics (genetic makeup, temperament, and pre‐ and perinatal factors) and environmental factors (family characteristics, parents and siblings, peers, and broader societal influences including media development). We introduce the Consortium of Individual Development (CID), which combines a series of integrated large-scale, multi-modal, longitudinal studies to take essential steps towards the ultimate goal of understanding and supporting this process.

## 1 Introduction

In order to function adequately in everyday life, being able to exert control over your emotions, behaviour, and cognition is crucially important. This ability is commonly referred to as self-regulation (Karoly, 1993). Developing adequate self-regulation is crucial for healthy development into adulthood, as is evidenced by associations between good self-regulation and a wide array of beneficial developmental outcomes such as better social, cognitive and emotional coping, and higher educational achievement (for an overview, see Rodriguez et al., 2005). However, more progress is needed towards, ultimately, a complete, integrated account of how self-regulation develops over time. Self-regulation and related constructs are widely studied but currently most studies are focused on either environment, or behaviour, or biology. As previous authors have noted, self-regulation results from interactions between such aspects and their interdependent development (e.g., Bell and Deater-Deckard, 2007; Casey and Caudle, 2013; Diamond and Aspinwall, 2003; Posner and Rothbart, 2000). Further, many studies are cross-sectional; those that do use a longitudinal design either study development within a relatively narrow age-range (Dennis et al., 2007; Lengua et al., 2015; Schoemaker et al., 2013), or do not include neuroimaging measures (Family Life Project, flp.fpg.unc.edu; The NICHD Study of Early Childcare, 2006; Dunedin birth cohort study, dunedinstudy.otago.ac.nz), or focus solely on adolescence (ABCD study, Volkow et al., 2018). More integration is needed between studies, different developmental periods, and developmental measures of self-regulation with repeated neuroimaging measurements. Longitudinal data using such an integrated approach is needed to provide a full account of the development of self-regulation.

In the current paper, we aim to provide a foundation for such research, focusing on a neurocognitive perspective. While we do not aim to review all factors related to self-regulation (such as important hormonal changes), we believe that the neurocognitive processes we focus on, despite only being a part of the whole story, may provide a way to organize our understanding of the very broad spectrum of different kinds of relevant influences. We hope to show how these influences could be seen to have a common endpoint in their effects on the adaptive development of certain neural functions causally proximate to observed cognitive and behavioural patterns of self-regulation. We will first outline the concept of self-regulation and recommend a clarification of terminology. Next, we present a concise overview of our current understanding of the development of self-regulation in terms of neural and behavioural development and related factors. This is followed by a critical assessment of the state of research on the development of self-regulation and a conceptual framework for future work. In so doing we will introduce the Consortium on Individual Development (CID), which aims to take essential steps in this direction. Work packages within CID concern more specific aspects of this complex and important process. Work package 1 (WP1), rooted in the Utrecht YOUth cohort, focuses on brain development in relation to behaviour, specifically on social competence and self-regulation and addresses their interrelation, and how associations might develop as a function of age, gender, genetic influences, and environmental exposures. WP2 aims to dissect the reason why not all children are equally responsive to variations in the social environment. It is based on the Leiden – CID Intervention Cohort, where large-scale experimental-longitudinal interventions of parent and peer behaviour allow for testing of which child characteristics shape the effect of (manipulated) environmental factors. WP3 focuses on the continuity of thriving (or failure to thrive) across three generations and uses information available in large existing and ongoing Dutch cohorts. Finally, WP4 complements the studies in work packages 1–3 with advanced mathematical modelling and animal research.

### 1.1 Self-regulation

Self-regulation has been defined as the ability to monitor and modulate our emotions, behaviour and cognition to allow us to achieve goals and adapt to changing circumstances (Berger et al., 2007; Karoly, 1993). It includes both automatic processes and effortful or deliberate control processes (Bridgett et al., 2015). Posner and Rothbart (2000) suggested that “understanding self-regulation is the single most crucial goal for advancing the understanding of development”, an observation that still resounds today (McClelland et al., 2018). Self-regulation comprises a set of abilities that prevents us from being left at the mercy of our environment and the reactive tendencies it provokes. Consequently, most theories suggest that self-regulation plays a role in various domains in life, both by facilitating positive behaviours and preventing undesirable ones. Indeed, a broad range of studies report positive associations between self-regulation abilities and school performance (Best et al., 2011), quality of social functioning (Eisenberg et al., 2000), and good mental health (Eisenberg et al., 2010). Moffitt et al. (2011) showed, based on data from a cohort of 1000 children from birth to adulthood, that self-control predicts better physical health, lower risk of substance dependence, better personal finances, and fewer criminal offending outcomes. Further, poor self-regulation has been linked to a variety of detrimental developmental outcomes that come with considerable costs for society: externalizing problem behaviour (Schoemaker et al., 2013), unhealthy behaviours such as those related to obesity and excessive weight gain (Caleza et al., 2016), poor academic performance (Bull et al., 2008), and violence and criminality (Vazsonyi et al., 2001).

Crucially, the emergence of self-regulation occurs in interaction with the environment in complex ways that result in positive or negative *developmental cascades* (Sapienza and Masten, 2011). These cascades describe the cumulative consequences for the many interactions between the developing system and its surroundings (Masten and Cicchetti, 2010). Consequently, low levels of self-regulation early on can impede development of self-regulation later. For example, very impulsive children tend to elicit negative reactions from others, which can limit their opportunities to practice and acquire self-regulation skills at older ages, subsequently creating a feedback loop of maladaptive behaviour and negative reactions (e.g., Rothbart and Bates, 1998). An essential environmental factor is early caregiving, which has been shown to be related to children’s developing capacities for self‐regulation (Kopp, 1982). For example, maternal positive affect during parent-child interaction early in development is associated with infants’ developing attention behavior (Swingler et al., 2017). Parents who interact by more positive affect, e.g., showing warm, accepting behaviour toward the child, may create an environment for the child in which the child feels comfortable and which promotes self-regulation. Results of a meta-analysis support the relationship between parenting and self-regulation in pre-schoolers (Karreman et al., 2006). The results showed that the way in which parents control their child is associated with the development of self-regulation. For example, positive control (e.g., limit-setting and the use of clear guidance and instructions) was positively related to self-regulation and negative control (e.g., coercive behaviours, critical comments or hostility) was negatively related to self-regulation. In other words, when parents use more positive guiding, teaching and encouragement towards their child, children seem to have higher levels of self-regulation (Karreman et al., 2006). The same results are revealed in older adolescents (aged 10–22) by a meta-analysis of more than 150 studies. Positive parenting and positive parent-child relationships are consistently, both concurrently and longitudinally, associated with better self-regulation, while negative parenting hampers self-regulation. In addition, and not surprisingly, child effects (higher adolescents’ self-regulation leading to more positive and less negative parenting) were also shown. Results appear to be very robust across gender and culture (Li et al., 2019).

Poor self-regulation can cascade into other problems such as lower academic achievement or comorbid disorders that have been associated with poor self-regulation, such as conduct disorder (Shannon et al., 2007). Importantly, in contrast to some other factors that relate to detrimental outcomes, such as social disadvantage and low intelligence, self-regulation may be considerably more malleable and therefore a better target for interventions (Diamond and Ling, 2016; Pandey et al., 2018; Piquero et al., 2016). An example of this is a recent study on Tools of the Mind (or *Tools*), a curriculum aimed at improving executive functions and the social context of the classroom (Diamond et al., 2019). Results in 315 kindergarten children showed improvements in self-control and executive function alongside better academic performance and better behaviour than students in more traditional classes.

One of the major difficulties in integrating data from the existing studies on the development of self-regulation involves the use of terminology. Many constructs have been linked to self-regulation, including effortful control (Rothbart and Bates, 1998), executive functioning (Garon et al., 2008), and self-control (Boutwell and Beaver, 2010); in the context of CID, the term “behavioural control” has been previously used to describe the ability to control emotions, behavior, impulses and adapt to rules. The often-interchangeable use of distinct yet related constructs has led to ‘conceptual clutter’ and therefore potentially impedes research progress (Morrisson and Grammer, 2016). Nigg (2017) has proposed a roadmap for a unified approach in developmental science by integrating the various concepts related to self-regulation into a single framework. Here, we adhere to this framework and apply it to the study of the development of self-regulation. We use “self-regulation” as a general umbrella term, as in Nigg (2017), who proposed it as a “domain-general construct that encompasses all self-regulation (including bottom up aspects)”, as well as in Zhou et al. (2012) who similarly use the term “self-regulation”. In our conceptual framework describing the development of self-regulation, we will closely follow the use of related terms recommended by Nigg (2017). First we use *executive functions,* which are defined as the set of hierarchically related top-down functions required for performing complex manipulations of information, under which we include the use of internal rules to govern behaviour; these functions are involved in more cognitive and behavioural processes than self-regulation alone, but low- and high-level executive functions play an important role in self-regulation. Second, we use *effortful control* to denote lower-level self-regulation, involving the use of relatively simple executive functions, such as attention or response inhibition. Effortful control is focused on responding to the immediate situation. Here, effortful control is used to cover both a trait and a type of process. Finally, we use the higher-level *strategic control* (“complex cognition and strategies” in Nigg, 2017) term, which refers to the use of higher-order executive functions to achieve more sophisticated forms of self-regulation, such as those involving planning for future events. Different levels of self-regulation arise at different developmental periods during the first two decennia of life.

## 2 Development of self-regulation

It is important to consider that self-regulation develops in interaction with a maturing brain. The state of the brain, for example the emergence of brain networks and the quality of their connections, dictates the possibilities and limits for self-regulation abilities at any given age. Vice versa, learning and adapting to new experiences affects brain development. Mapping these intricate interactions will shed light on *how* environmental factors and child characteristics influence the development of self-regulation. As such, including neuroimaging measures in a longitudinal study design may improve our understanding of how self-regulation develops. In CID, studying the development of self-regulation is coupled with extensive neuroimaging, ranging from ultra-sounds at 20 weeks of pregnancy, foetal and post-natal functional and structural MRIs, EEG measurements from 5 months to 6 years, and detailed functional and structural MRIs from age 6 onwards. Importantly, CID includes an animal cohort (WP4), which allows us to investigate in more detail the neural developmental processes underlying self-regulation. Moreover, as we gain more insight into these processes in animals, we can better inform research questions for humans. Likewise, the human data we collect will prompt specific questions that can be addressed using animals.

Brain maturation is not a simple linear process of growth. Maturation occurs in distinct developmental periods which can be distinguished by the onset or end of specific neural processes. Indeed, brain development is characterised by a tremendous growth of both gray and white matter during the first two years, which is then followed by periods of slower volume increase and ultimately decrease in gray matter volume (Giedd et al., 1999; Wierenga et al., 2014). During adolescence, the pattern of maturation varies spatiotemporally over the brain, with subcortical regions related to motivation maturing before prefrontal development (Casey, 2015; Casey et al., 2008; Gladwin et al., 2011). These brain changes facilitate the type of skill acquisition that occurs in each developmental period. During subsequent periods, more advanced learning and brain maturation processes build on previous changes to support further refinement of these skills (Casey et al., 2019). Vice versa, experience and training of these new skills affect the same brain maturation processes. However, and as mentioned before, although the major developmental periods in brain maturation have been charted (Gilmore et al., 2018), and there are ideas about how the development of self-regulation and brain maturation are related, there are almost no data to support such ideas. We will briefly discuss the development of self-regulation and mention the most important brain maturation processes for three developmental periods: infancy and early childhood, childhood, and adolescence. Although many more developmental periods could potentially be distinguished, these periods figure prominently in the existing literature on both self-regulation and brain development. These periods also align with questionnaires on self-regulation that are being used throughout CID. As these questionnaires are similar across all cohorts that together make up CID, experimental measures can be integrated across studies and age-ranges since they can be anchored to these questionnaires.

### 2.1 Self-regulation and brain maturation in infancy and early childhood: Effortful control

The study of self-regulation during infancy and early childhood builds heavily on the pivotal work by Rothbart and colleagues (Rothbart, 1981), who first coined the term effortful control. In the framework proposed by Nigg (2017), effortful control at this age refers to the top-down control over bottom-up processes for purposes of self-regulation. There is consensus that self-regulation shifts during the early years of development from a pattern of predominantly reactive responding to external stimuli that is supported by parents in infancy, towards deliberate control of internal states in early childhood (Rothbart et al., 1990). During infancy, the parent initially acts as an external regulator (Calkins and Fox, 2002; Kopp, 1982). For example, attention behavior in newborns is controlled externally and is depended on the properties of the stimuli of the environment. During the first year of life, the emergence of voluntary control of behaviors occurs with the development of an executive system within the frontal cortex (Calkins and Fox, 2002). The transition from external to internal regulation of behavior is of great importance (Kochanska et al., 2001). However, much of the behavior of a child continues to develop in the context of a parent-child dyad (Calkins and Fox, 2002; Kopp, 1982). This has been recognized in research on the effect of parenting on the development of children for more than half a century (Belsky and de Haan, 2011). Many studies revealed associations between maternal behaviors during infancy and performance on executive functioning tasks later in childhood (e.g., Cuevas et al., 2014; Kraybill and Bell, 2013). In CID attentional control in early childhood is tested via eye movement measures (WP1). Further, the role of parents may well be essential already in these early interactions, which may be related to intergenerational patterns of behaviour (WP3).

The low-level executive functions that are fundamental to early-life self-regulation begin to emerge over the first years of life (Eisenberg and Zhou, 2016; Rothbart et al., 2003). Importantly, Rothbart and colleagues (Rothbart et al., 2003) excluded high-level executive functions such as planning, problem solving, information processing and cognitive flexibility in their definition of effortful control. This is consistent with the finding that in very young children, executive functions hardly extend beyond temporarily overcoming a stimulus-driven response (Garon et al., 2008) and thus are low-level rather than high-level. This is in line with the notion that self-regulation involves only effortful control and associated low-level executive functions in earlier but not later stages of development, when age-appropriate self-regulation could additionally involve different and more complex cognitive processes.

Executive functions underlying self-regulation, e.g., inhibitory control and attention regulation, depend on sufficiently progressed brain development (Garon et al., 2008). The rise of more complex self-regulation is paralleled by the development of the orienting-attention network that enables children to orient to stimuli and to shift attention from one stimulus to another (Posner and Rothbart, 2018), and subsequently the executive attention network (Posner and Rothbart, 2007). The latter network is thought to become more influential after two years of age and continues to develop well until early adulthood (Posner et al., 2014). More specifically, several cross-sectional imaging studies have reported positive associations between measures of brain functional connectivity (as measured with EEG) and precursors of self-regulation, such as object permanence (Bell and Fox, 1997; Cuevas et al., 2012), working memory (Bell and Wolfe, 2007), attentional control (Whedon et al., 2016), and inhibitory control (Broomell et al., 2019; Swingler et al., 2011) in infancy and early childhood.

Early environmental experiences are closely related to brain development, and it seems likely that the association between parenting behaviors and child cognitive development is a result of the interplay between genetic factors, brain development and the social environment (De Bellis, 2005). Both maternal behaviors and frontal brain activity measured with EEG at 10 months old predicted perfomance on executive tasks at 3 and 5 years old (Kraybill and Bell, 2013). Variation in parenting behaviors is predictive for brain development during the first years of life (Bernier et al., 2016). For example, maternal intrusiveness when infants are 5 months is related to brain activity at the left medial frontal location and attention regulation at 10 months (Swingler et al., 2017). These findings suggest that maternal behavior affects brain development related to the development of attention behavior. Results of a previous study revealed that infants had higher frontal brain activity when their parents interacted by more positive affect (Bernier et al., 2016). These results suggest that the quality of maternal behavior is related to increases in frontal brain activity during infancy. However, results of the study conducted by Swingler et al. (2017) showed that maternal positive affect was not significantly associated with frontal brain activity. Therefore, more research is needed to examine the relationship between parenting behaviors, brain development and (precursors of) self-regulation.

In CID, EEG recordings of brain activation are being made during infancy and early childhood from 5 months up until age 7 (WP1). From 7 years onwards, functional and structural MRI is recorded so that brain networks can be investigated both in terms of spatial and temporal components (WP1 and WP2).

### 2.2 Self-regulation and brain maturation in childhood: Higher-level executive functions

By the time children go to school, they are facing increasingly complex tasks and situations that call for more advanced levels of self-regulation. As noted above, *executive functions* play a role in self-regulation, and these functions become increasingly taxed during the (pre-) school period (Garon et al., 2008). Indeed, many strategies for self-regulation, for example inhibiting the tendency to look at or touch a treat when instructed to wait (i.e. delayed gratification task, Casey et al., 2011; Mischel et al., 2011), seem clearly related to the employment of executive functions to the aim of self-regulation as conceptualized by Nigg (2017). Children need to develop and hone high-level executive functions, such as planning, problem solving, information processing and cognitive flexibility (Rueda et al., 2005). These high-level executive functions build on the integration of the low-level executive functions developed in infancy and preschool years (Diamond, 2013). Unlike the improvements in preschool years, however, these later refinements seem to involve quantitative improvements in accuracy, perhaps due to an increasing efficiency in overriding prepotent responses. In CID, several tests are administered which challenge children to employ strategies to handle distractors while maintaining focus on the goal, (WP1, 2 and 3). Such tests shed light on the child’s ability to tackle such a challenge, including not only cognitive aspects but also how children and parents interact when such a challenge arises.

The refinement in behaviour is paralleled by distinct neural changes throughout this developmental period, shifting from the increasing volume of regions of the brain to more subtle changes. Whereas brain development during early life can be broadly characterized by volume expansion and neuron growth and /synapse formation, during childhood gray matter volume starts to shrink (Giedd et al., 1999; Wierenga et al., 2014). Indeed, after the age of five, cortical thickness begins to decrease. The speed at which this occurs varies for different brain regions (Gogtay and Thompson, 2010). Nevertheless, the brain continues to expand, mainly driven by the myelination of white-matter nerve fibres (Paus, 2010). Together with synaptic pruning, and the fact that this pruning is commonly associated with learning (Craik and Bialystok, 2006), it is thought that these processes combine to form efficient brain networks that support the shift from low-level to high-level executive functions. Indeed, the gray matter changes coincide with increases in myelination. Although the peak of myelination occurs during the first year of life, it continues into young adulthood, especially in some cortical areas of the brain (Fields, 2008). This raises the possibility that myelin, together with dynamic cortical changes, plays an important role in optimizing information processing through experience. For example, it is thought that the development of alerting, orienting, and strategic control during childhood is supported by the improvement in the efficiency of long-range connections in the supporting networks (Posner et al., 2016). Data in support of these ideas come from a recent EEG study which showed that in children between 7 and 9 years of age the general pattern of maturation consisted of an increase in long-distance connections with posterior cortical regions and a decrease in short connections within prefrontal cortical areas, and that this pattern was related to scores on effortful control questionnaires (Knyazev et al., 2017).

Brain development during this phase of childhood, as does all brain development, occurs in interaction with the environment (Belsky and de Haan, 2011; Greenough et al., 1987). For example, neurons that are actively stimulated through environmental experiences are strengthened and neurons that are rarely or not activated will be eliminated. This competitive interaction between neuronal connections is an important process of brain development (Greenough et al., 1987). Therefore, environmental experiences can maintain or enhance normal child development, or at the same time, adversely affect it (Belsky and de Haan, 2011). Not only brain functioning, but also brain structure and connectivity can be adversely affected by negative environmental experiences. For example, results of previous studies show an association between maltreatment during childhood and abnormalities in the cortical network (Teicher et al., 2014), smaller corpus callosum areas (Sheridan et al., 2012) and diminished BOLD response of striatal regions (Mehta et al., 2010).

### 2.3 Self-regulation and brain maturation in adolescence: completing strategic control

During adolescence, the various executive functions start to become integrated to support high-level strategic control. Strategic control requires the goal-directed coordination of previously acquired low- and high-level executive functions such as working memory, inhibition, mental shifting, and information processing (Best et al., 2011; Friedman et al., 2008). Strategic control is the level of self-regulation that needs to be established during adolescence. For example, while children at the end of childhood can inhibit prepotent responses, a lower-level executive function, they become much more skilled in inhibitory control during adolescence (Vink et al., 2014b). This improvement is associated with the rise of proactive response strategies that allow for a more efficient processing by engaging inhibitory functions prior to having to stop your response (Zandbelt and Vink, 2010). As such, the improvement in self-regulation in adolescence seems to be due to the effective integration and coordination of executive functions.

In the brain, the further development of self-regulation, and thus the rise of strategic control, has been theorized to co-occur with the vast improvement of the quality of connections between cortical and subcortical regions (Casey et al., 2019; Cools, 2011; Padmanabhan et al., 2011; Vink et al., 2014b). This is facilitated at the onset of adolescence by the increase in myelination of white-matter tracts connecting these regions (Asato et al., 2010; De Leeuw et al., 2017; Ladouceur et al., 2012), allowing for faster and more precise neural signalling. Such anatomical changes directly affect brain function and hence behaviours linked to improvements in self-regulation. For example, functional MRI data have shown that the shift from reactive to a more planning-based proactive inhibition strategy was paralleled in the brain by increased frontal activation as well as increased functional connectivity between frontal and subcortical regions (Vink et al., 2014b).

However, while self-regulation and frontal-subcortical connectivity ultimately increase during the transition from child- to adulthood (Casey, 2015; Somerville and Casey, 2010), adolescence is also characterized by non-linear changes in sensitivity to salient, motivating stimuli. This shows strategic control extending beyond effortful control to include complex cognition, including cognition involving emotion and motivation. Adolescence is typically associated with behaviours such as increased risk taking, impulsivity, and heightened sensitivity to social cues (van Duijvenvoorde et al., 2016), associated with adolescent-specific peaks in activation in striatal reward regions (Braams et al., 2015; Van Leijenhorst et al., 2010; Vink et al., 2014a). It has been hypothesized that these indicators of reduced self-regulation capacity in adolescence, most notably in the presence of incentives, is related to a developmental, transient imbalance between frontal lobe control and subcortical reward processing (Geier et al., 2010; Gladwin et al., 2011; Hoogendam et al., 2013; Padmanabhan et al., 2011). This is thought to be a result of regional differences in speed of maturation across the brain, with the frontal cortex thought to develop slowest (Lenroot and Giedd, 2006), yet no longitudinal data exist to test the hierarchical changes in frontal-striatal circuitry development in relation to self-regulation during adolescence. In order to facilitate the collection of longitudinal data on the development of these networks, CID records structural MRI data to visualize anatomical connectivity and gray/white-matter developments, as well as functional MRI data during cognitive tasks and during resting-state (WP1 and WP2).

## 3 Towards an integrated account of the development of self-regulation

Despite the large body of literature on effortful control, executive functions, strategic control, and other related concepts in children and adolescents, there is no truly developmental account of self-regulation across childhood, let alone from childhood into adolescence (Best and Miller, 2010). Such an account would need to integrate child characteristics and environmental factors across the entire developmental period to explain the mechanisms underlying developmental changes, explain the long-term consequences of suboptimal development, and suggest appropriate interventions.

McClelland et al. (2018) recently voiced their concern about this when they stated that “the study of self-regulation lacks integration across the life span”. Furthermore, the lack of an integrated account of the development of self-regulation seems worrisome, especially given the potentially tremendous beneficial impact of uncovering developmental trajectories from infancy to adulthood, both in terms of typical and atypical development.

### 3.1 Three reasons why we don’t yet have an integrated developmental account

The first reason for the lack of integration across studies is the fact that self-regulation is conceptualized, labelled, and measured in many different ways. This hinders the integration of data from different studies and groups, even when they focus on the same developmental period, let alone data from studies on different developmental periods. The second reason is the lack of integration across different developmental periods. Most (imaging) studies focus on a particular developmental period, such as adolescence or the preschool period. As a result, the available developmental theories and models are primarily based on data taken from a plethora of relatively small studies, either cross-sectional or longitudinal in nature. The third reason is the lack of integration of developmental measures of self-regulation with repeated neuroimaging measurements. Indeed, the few studies that do follow-up children over a long period of time (Family Life Project, flp.fpg.unc.edu; The NICHD Study of Early Childcare, 2006; Dunedin birth cohort study, dunedinstudy.otago.ac.nz) do not include neuroimaging measures. Although there are plausible theories linking brain development to the establishment of self-regulation, as discussed in the previous section, many of the ideas regarding brain development are largely based on findings from comparative research with monkeys, adult neuroimaging studies, or symptoms in clinical patients with lesions in certain brain areas (Spear, 2000). As a consequence of the lack of longitudinal data linking brain maturation to the development of self-regulation, we do not know how environmental factors and child characteristics affect the interplay between brain development and the development of self-regulation.

### 3.2 Where do we want to go? Working towards an integrated account of the development of self-regulation

What is ultimately needed is an integrated approach to the study of self-regulation, in which longitudinal data on brain, behaviour and environment are all taken into consideration. In that way, we can map the dynamics of typical neural and behavioural development, understand causal relationships in terms of specific, well-defined mechanisms, unravel developmental cascades and engineer interventions for cases where development goes awry. Poor self-regulation in childhood has been linked to a variety of problems later in life. An integrated account of the development of self-regulation should help identify factors that affect the development of self-regulation and offer insights into how the effects of early problems can be countered or minimized.

**Fig. 1:**
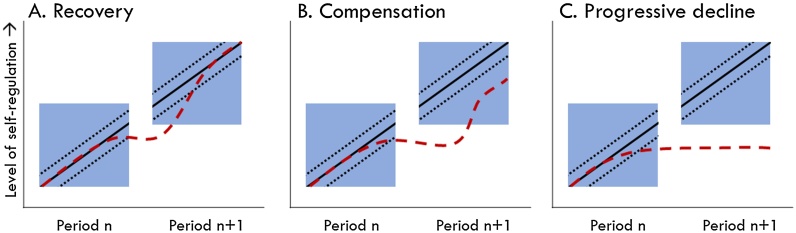
Hypothetical schematic representation of developmental pathways of self-regulation within development periods (indicated by blue squares). The solid black line denotes typical development of self-regulation plotted against age, for subsequent developmental periods. Two consecutive periods are shown. The dotted black lines indicate the boundaries of typical development. The dotted red line represents a hypothetical atypical development of self-regulation, that shows (A) recovery to typical levels, (B) compensation, or (C) relative progressive decline across development. (For interpretation of the references to colour in this figure legend, the reader is referred to the web version of this article).

This is of particular relevance as it has been demonstrated that the development of self-regulation is, to a certain degree, malleable, suggesting that not all children that display poor self-regulation will necessarily have poor self-regulation in adulthood (Diamond and Ling, 2016; Pandey, Hale, Das, Goddings, Blakemore, and Viner, 2018; Piquero et al., 2016). Hypothetical scenarios following a problem at a point in the development of self-regulation are shown in Fig. 1. In some cases, recovery to (near)normal levels may occur due to developmental processes which onset in a new developmental period (Fig. 1A). Indeed, it has been shown that some children with reduced levels of self-regulation early on can catch up by means of accelerated growth (Moilanen et al., 2010), which can be facilitated by training (Chang et al., 2014). These processes may occur in the environment (for example, starting your school career), in the brain (for example, myelination starts to more effectively facilitate neural signalling between brain regions), or any interaction between them. We need to identify such factors that support recovery to typical levels of self-regulation. Alternatively, recovery may not be possible, but rather the problems that occurred may be compensated to some extent by integrating multiple lower-level skills later on (Fig. 1B). We need to understand in what circumstances such compensation is possible. Compensation can also occur in the brain by means of additional compensatory activations underlying (near-to) normal behavioural performance. Overactivation suggestive of such compensation has been observed, for example, in children at high familial risk for schizophrenia (Vink et al., 2015), or those with ADHD, autism, or developmental disorders in general (Cortese et al., 2012; Fassbender and Schweitzer, 2006; Johnson, 2012; Johnson et al., 2015). However, as suggested by Masten and colleagues (Masten and Cicchetti, 2010), early problems may instead trigger a negative developmental cascade that causes relative progressive worsening of self-regulatory capabilities (Fig. 1C). Such a scenario is also consistent with the idea that poor skill development early on has the greatest impact on functioning later on in life, as the skills that develop early are fundamental to all subsequent skills.

As such, it is essential to understand when and why different patterns of long-term consequences of dysregulation emerge. This requires the integration of environmental measurements with repeated behavioural, cognitive and neuroimaging assessments of self-regulation research across the various developmental periods, from infancy into early adulthood.

It is thus crucial that longitudinal brain development data is incorporated in developmental accounts of self-regulation to uncover multi-directional relationships between environment, brain processes and the emergence and shaping of self-regulation over time. Although there is ample knowledge on brain development in general, and on the impact on behaviour of learning and experience in a broad sense, there is a lack of studies and theories that aim to link these factors. Nature and nurture are inextricably interlinked (Sameroff, 2010): The adaptive mechanisms of the brain must be ready to permit the possibility of learning new skills, including self-regulation, and the right learning experiences must be provided to it for this potential to be realized. Therefore, understanding why certain children are better able than others in learning new skills requires incorporating behavioural and environmental information with data about the brain. The state of the brain depends in part on biological maturation processes, but also on prior learning from previous behaviour and environmental stimulation. Subsequently, the state of the brain will determine behaviour and thus affect environmental feedback and the ability to learn from it (Driemeyer et al., 2008).

This embedding of self-regulation in learning and reinforcement processes has been made explicit in some models of dual processing and working memory (Gladwin et al., 2011; Gladwin and Figner, 2014; Hazy et al., 2006; Pessoa, 2009). In normal development, changes in the child’s daily life go hand in hand with what the child is ready for and requires for further development. For instance, the social demands of going to school would be impossible to deal with if the brain was not ready, but conversely without a sufficiently rich social context the neural maturation will not be correctly finetuned during sensitive periods (Hensch, 2004; Newport, 1990; Penhune and de Villers-Sidani, 2014). Conversely, if a child, for instance, learns that waiting too long to react results in being verbally abused or in siblings taking away toys or food, he or she will learn not to delay. In their individual context, this is adaptive learning and does not reflect unpreparedness or delayed brain development to learn different behavioural responses. We suggest that such problems are best understood from the perspective of executive functions as reinforced responses, in line with both radical behaviourist (Heward and Cooper, 1992) and neuroscientific (Hazy et al., 2007; Pessoa, 2009) perspectives. The relationship between reinforced executive functions and self-regulation is a core part of the definition of “R^3^-reflectivity” in the Reprocessing/Reentrance and Reinforcement model of Reflectivity (Gladwin et al., 2011; Gladwin and Figner, 2014). This model evolved from earlier dual-system models of distinct impulsive versus reflective processes or systems and subsequent criticisms of such models (Keren and Schul, 2009; Pfeifer and Allen, 2012). One important element of the model is its emphasis on how executive functions must be selected based on emotionally relevant outcomes predicted due to prior learning experiences provided by the individual’s environment (Gladwin et al., 2019). Further, reflective processing and self-regulation are argued to emerge as a function of time - in the sense of the hundreds of milliseconds following a stimulus - due to the different temporal dynamics of different cognitive processes involved in (cognitive) response selection, rather than there being a separation and competition between sets of reflective and impulsive processes or brain regions. For instance, reinforcement learning networks in the basal ganglia are fundamentally necessary for coordinated activation in the cortex (Hazy et al., 2007; Lawrence, 2000; Samejima and Doya, 2007), rather than there being a kind of “subcortical subconscious” competing with the “rational” cortex. Similarly, there is no conflict between considering neural versus environmental factors: It naturally follows from a view such as ours that both of these are part of the full story and inherently require each other, due to the adaptive nature of the neural processes of interest. The degree of self-regulation expressed by an individual would therefore be expected to arise from the causal interactions between neural maturation and preparedness, social context, family, school, and all sorts of child characteristics and environmental factors; rather than from a simple presence or absence of a “deficit” or “imbalance”. As future research into such models develops, more detailed knowledge will be acquired on when certain kinds of neural preparedness tend to arise, which kind of experiences provide optimal versus suboptimal learning opportunities for the newly developed networks, and why deficits persist or dissipate in subsequent phases.

## 4 Conclusion: moving towards clinically relevant theory

The Consortium on Individual Development (CID), by using the same instruments in all cohorts, addresses a range of essential factors in the development of self-regulation and allows for the analysis of the same concept - self-regulation - measured in a comparable way, in different cohorts tapping into different environmental factors and brain and behavioural measures throughout childhood and adolescence. The data collected in the CID cohorts hence provide a first step towards an integrated account of the development of self-regulation, by [1] sharing our conceptual framework to integrate concepts and terms related to how self-regulation develops via key (neurocognitive) processes, [2] integrating longitudinal measures of a wide range of child characteristics and environmental factors (WP1, WP2 and WP3), [3], developing animal models that can start to specify in more detail the neurocognitive mechanisms involved in self-regulation (WP4), and [4] integrating developmental measures of self-regulation with repeated neuroimaging measurements (WP1 and WP2).

As the developmental account of self-regulation itself matures, we expect clinical applications to grow along with it. First, once the specific processes necessary for progression are known, early detection of aberrant processes may become possible. Second, an improved understanding of the developmental processes necessary to arrive at adaptive self-regulation will point to specific interventions. These interventions would be targeted at the processes relevant for a given state of development and could involve a logical, theory-driven combination of approaches. For example, if a cognitive process necessary for future self-regulation is lacking, this could be targeted via, e.g., training inhibitory functions in combination with transient electrical brain stimulation of relevant brain regions (Ditye et al., 2012). At the same time, attention would be paid to reinforcing any trained cognitive skills, in particular within the child’s social context, so they become part of a repertoire of available cognitive responses. There would thus be no artificial separation between different kinds of interventions; rather, it would become clear exactly why and how different approaches must, logically, be integrated. We hope that CID via its work packages presented here, similar studies, and studies within this framework of understanding development, will take steps towards this ultimate goal of effective, targeted, theory-driven and evidence-based intervention.

## Declaration of Competing Interest

None.

## References

[bib0005] Asato M.R., Terwilliger R., Woo J., Luna B. (2010). White matter development in adolescence: a DTI study. Cereb. Cortex.

[bib0010] Bell M.A., Deater-Deckard K. (2007). Biological systems and the development of self-regulation: integrating behavior, genetics, and psychophysiology. J. Dev. Behav. Pediatr..

[bib0015] Bell M.A., Fox N.A. (1997). Individual differences in object permanence performance at 8 months: locomotor experience and brain electrical activity. Dev. Psychobiol..

[bib0020] Bell M.A., Wolfe C.D. (2007). Changes in brain functioning from infancy to early childhood: evidence from EEG power and coherence working memory tasks. Dev. Neuropsychol..

[bib0025] Belsky J., De Haan M. (2011). Annual research review: parenting and children’s brain development: the end of the beginning. J. Child Psychol. Psychiatry.

[bib0030] Berger A., Kofman O., Livneh U., Henik A. (2007). Multidisciplinary perspectives on attention and the development of self-regulation. Prog. Neurobiol..

[bib0035] Bernier A., Calkins S.D., Bell M.A. (2016). Longitudinal associations between the quality of mother–infant interactions and brain development across infancy. Child Dev..

[bib0040] Best J.R., Miller P.H. (2010). A developmental perspective on executive function. Child Dev..

[bib0045] Best J.R., Miller P.H., Naglieri J.A. (2011). Relations between executive function and academic achievement from ages 5 to 17 in a large, representative national sample. Learn. Individ. Differ..

[bib0050] Boutwell B.B., Beaver K.M. (2010). The intergenerational transmission of low self-control. J. Res. Crime Delinq..

[bib0055] Braams B.R., van Duijvenvoorde A.C.K., Peper J.S., Crone E.A. (2015). Longitudinal changes in adolescent risk-taking: a comprehensive study of neural responses to rewards, pubertal development, and risk-taking behavior. J. Neurosci..

[bib0060] Bridgett D.J., Burt N.M., Edwards E.S., Deater-Deckard K. (2015). Intergenerational transmission of self-regulation: a multidisciplinary review and integrative conceptual framework. Psychol. Bull..

[bib0065] Broomell A.P.R., Savla J., Bell M.A. (2019). Infant electroencephalogram coherence and toddler inhibition are associated with social responsiveness at age 4. Infancy.

[bib0070] Bull R., Espy K.A., Wiebe S.A. (2008). Short-term memory, working memory, and executive functioning in preschoolers: longitudinal predictors of mathematical achievement at age 7 years. Dev. Neuropsychol..

[bib0075] Caleza C., Yañez-Vico R.M., Mendoza A., Iglesias-Linares A. (2016). Childhood obesity and delayed gratification behavior: a systematic review of experimental studies. J. Pediatr..

[bib0080] Calkins S.D., Fox N.A. (2002). Self-regulatory processes in early personality development: a multilevel approach to the study of childhood social withdrawal and aggression. Dev. Psychopathol..

[bib0085] Casey B.J. (2015). Beyond simple models of self-control to circuit-based accounts of adolescent behavior. Annu. Rev. Psychol..

[bib0090] Casey B.J., Caudle K. (2013). The teenage brain: self control. Curr. Dir. Psychol. Sci..

[bib0095] Casey B.J., Jones R.M., Hare T. (2008). The adolescent brain. Ann. N. Y. Acad. Sci..

[bib0100] Casey B.J., Somerville L.H., Gotlib I.H., Ayduk O., Franklin N.T., Askren M.K., Jonides J., Berman M.G., Wilson N.L., Teslovich T., Glover G., Zayas V., Mischel W., Shoda Y. (2011). Behavioral and neural correlates of delay of gratification 40 years later. Proc. Natl. Acad. Sci. U. S. A..

[bib0105] Casey B.J., Heller A.S., Gee D.G., Cohen A.O. (2019). Development of the emotional brain. Neurosci. Lett..

[bib0110] Chang H., Shaw D.S., Dishion T.J., Gardner F., Wilson M.N. (2014). Direct and indirect effects of the family check-up on self-regulation from toddlerhood to early school-age. J. Abnorm. Child Psychol..

[bib0115] Cools R. (2011). Dopaminergic control of the striatum for high-level cognition. Curr. Opin. Neurobiol..

[bib0120] Cortese S., Kelly C., Chabernaud C., Proal E., Di Martino A., Milham M.P., Castellanos F.X. (2012). Toward systems neuroscience of ADHD: a meta-analysis of 55 fMRI studies. Am. J. Psychiatry.

[bib0125] Craik F.I.M., Bialystok E. (2006). Cognition through the lifespan: mechanisms of change. Trends Cogn. Sci..

[bib0130] Cuevas K., Swingler M.M., Bell M.A., Marcovitch S., Calkins S.D. (2012). Measures of frontal functioning and the emergence of inhibitory control processes at 10 months of age. Dev. Cogn. Neurosci..

[bib0135] Cuevas K., Deater‐Deckard K., Kim‐Spoon J., Watson A.J., Morasch K.C., Bell M.A. (2014). What’s mom got to do with it? Contributions of maternal executive function and caregiving to the development of executive function across early childhood. Dev. Sci..

[bib0140] De Bellis M.D. (2005). The psychobiology of neglect. Child Maltreat..

[bib0145] De Leeuw M., Bohlken M.M., Mandl R.C.W., Hillegers M.H.J., Kahn R.S., Vink M. (2017). Changes in white matter organization in adolescent offspring of schizophrenia patients. Neuropsychopharmacology.

[bib0150] Dennis T.A., Brotman L.M., Huang K.-Y., Gouley K.K. (2007). Effortful control, social competence, and adjustment problems in children at risk for psychopathology. J. Clin. Child Adolesc. Psychol..

[bib0155] Diamond A. (2013). Executive functions. Annu. Rev. Psychol..

[bib0160] Diamond L.M., Aspinwall L.G. (2003). Emotion regulation across the life span: an integrative perspective emphasizing self-regulation, positive affect, and dyadic processes. Motiv. Emot..

[bib0165] Diamond A., Ling D.S. (2016). Conclusions about interventions, programs, and approaches for improving executive functions that appear justified and those that, despite much hype, do not. Dev. Cogn. Neurosci..

[bib0170] Diamond A., Lee C., Senften P., Lam A., Abbott D. (2019). Randomized control trial of tools of the mind: marked benefits to kindergarten children and their teachers. PLoS One.

[bib0175] Ditye T., Jacobson L., Walsh V., Lavidor M. (2012). Modulating behavioral inhibition by tDCS combined with cognitive training. Exp. Brain Res..

[bib0180] Driemeyer J., Boyke J., Gaser C., Büchel C., May A. (2008). Changes in gray matter induced by learning—revisited. PLoS One.

[bib0185] Eisenberg N., Fabes R.A., Guthrie I.K., Reiser M. (2000). Dispositional emotionality and regulation: their role in predicting quality of social functioning. J. Pers. Soc. Psychol..

[bib0190] Eisenberg N., Spinrad T.L., Eggum N.D. (2010). Emotion-related self-regulation and its relation to children’s maladjustment. Annu. Rev. Clin. Psychol..

[bib0195] Eisenberg N., Zhou Q., Griffin J.A., McCardle P., Freund L. (2016). Conceptions of executive function and regulation: when and to what degree do they overlap?. Executive Function in Preschool-Age Children: Integrating Measurement, Neurodevelopment, and Translational Research.

[bib0200] Fassbender C., Schweitzer J.B. (2006). Is there evidence for neural compensation in attention deficit hyperactivity disorder? A review of the functional neuroimaging literature. Clin. Psychol. Rev..

[bib0205] Fields R.D. (2008). White matter in learning, cognition and psychiatric disorders. Trends Neurosci..

[bib0210] Friedman N.P., Miyake A., Young S.E., DeFries J.C., Corley R.P., Hewitt J.K. (2008). Individual differences in executive functions are almost entirely genetic in origin. J. Exp. Psychol. Gen..

[bib0215] Garon N., Bryson S.E., Smith I.M. (2008). Executive function in preschoolers: a review using an integrative framework. Psychol. Bull..

[bib0220] Geier C.F., Terwilliger R., Teslovich T., Velanova K., Luna B. (2010). Immaturities in reward processing and its influence on inhibitory control in adolescence. Cereb. Cortex.

[bib0225] Giedd J.N., Blumenthal J., Jeffries N.O., Castellanos F.X., Liu H., Zijdenbos A., Paus T., Evans A.C., Rapoport J.L. (1999). Brain development during childhood and adolescence: a longitudinal MRI study. Nat. Neurosci..

[bib0230] Gilmore J.H., Knickmeyer R.C., Gao W. (2018). Imaging structural and functional brain development in early childhood. Nat. Rev. Neurosci..

[bib0235] Gladwin T.E., Figner B., Wilhelms E., Reyna V.F. (2014). “Hot” cognition and dual systems: introduction, criticisms, and ways forward. Frontiers of Cognitive Psychology Series: Neuroeconomics, Judgment and Decision Making.

[bib0240] Gladwin T.E., Figner B., Crone E.A., Wiers R.W. (2011). Addiction, adolescence, and the integration of control and motivation. Dev. Cogn. Neurosci..

[bib0245] Gladwin T.E., Figner B., Vink M. (2019). Anticipation-specific reliability and trial-to-trial carryover of anticipatory attentional bias for threat. J. Cogn. Psychol..

[bib0250] Gogtay N., Thompson P.M. (2010). Mapping gray matter development: implications for typical development and vulnerability to psychopathology. Brain Cogn..

[bib0255] Greenough W.T., Black J.E., Wallace C.S. (1987). Experience and brain development. Child Dev..

[bib0260] Hazy T.E., Frank M.J., O’Reilly R.C. (2006). Banishing the homunculus: making working memory work. Neuroscience.

[bib0265] Hazy T.E., Frank M.J., O’Reilly R.C. (2007). Towards an executive without a homunculus: computational models of the prefrontal cortex/basal ganglia system. Philos. Trans. R. Soc. Lond., B, Biol. Sci..

[bib0270] Hensch T.K. (2004). Critical period regulation. Annu. Rev. Neurosci..

[bib0275] Heward W.L., Cooper J.O. (1992). Radical behaviorism: a productive and needed philosophy for education. J. Behav. Educ..

[bib0280] Hoogendam J.M., Kahn R.S., Hillegers M.H.J., Van Buuren M., Vink M. (2013). Different developmental trajectories for anticipation and receipt of reward during adolescence. Dev. Cogn. Neurosci..

[bib0285] Johnson M.H. (2012). Executive function and developmental disorders: the flip side of the coin. Trends Cogn. Sci..

[bib0290] Johnson M.H., Jones E.J.H., Gliga T. (2015). Brain adaptation and alternative developmental trajectories. Dev. Psychopathol..

[bib0295] Karoly P. (1993). Mechanisms of self-regulation: a systems view. Annu. Rev. Psychol..

[bib0300] Karreman A., Van Tuijl C., van Aken M.A., Deković M. (2006). Parenting and self‐ regulation in preschoolers: a meta‐analysis. Infant Child Dev..

[bib0305] Keren G., Schul Y. (2009). Two is not always better than one. Perspect. Psychol. Sci..

[bib0310] Knyazev G.G., Savostyanov A.N., Bocharov A.V., Slobodskaya H.R., Bairova N.B., Tamozhnikov S.S., Stepanova V.V. (2017). Effortful control and resting state networks: a longitudinal EEG study. Neuroscience.

[bib0315] Kochanska G., Coy K.C., Murray K.T. (2001). The development of self‐regulation in the first four years of life. Child Dev..

[bib0320] Kopp C.B. (1982). Antecedents of self-regulation: a developmental perspective. Dev. Psychol..

[bib0325] Kraybill J.H., Bell M.A. (2013). Infancy predictors of preschool and post‐kindergarten executive function. Dev. Psychobiol..

[bib0330] Ladouceur C.D., Peper J.S., Crone E.A., Dahl R.E. (2012). White matter development in adolescence: the influence of puberty and implications for affective disorders. Dev. Cogn. Neurosci..

[bib0335] Lawrence A. (2000). Error correction and the basal ganglia: Similar computations for action, cognition and emotion?. Trends Cog. Sci..

[bib0340] Lengua L.J., Moran L., Zalewski M., Ruberry E., Kiff C., Thompson S. (2015). Relations of growth in effortful control to family income, cumulative risk, and adjustment in preschool-age children. J. Abnorm. Child Psychol..

[bib0345] Lenroot R.K., Giedd J.N. (2006). Brain development in children and adolescents: insights from anatomical magnetic resonance imaging. Neurosci. Biobehav. Rev..

[bib0350] Li J.-B., Willems Y.E., Stok F.M., Deković M., Bartels M., Finkenauer C. (2019). Parenting and self-control across early to late adolescence: a three-level meta-analysis. Perspect. Psychol. Sci..

[bib0355] Masten A.S., Cicchetti D. (2010). Developmental cascades. Dev. Psychopathol..

[bib0360] McClelland M., Geldhof J., Morrison F., Gestsdóttir S., Cameron C., Bowers E., Duckworth A., Little T., Grammer J., Halfon N., Forrest C.B., Lerner R.M., Faustman E.M. (2018). Self-regulation. Handbook of Life Course Health Development.

[bib0365] Mehta M.A., Gore-Langton E., Golembo N., Colvert E., Williams S.C., Sonuga- Barke E. (2010). Hyporesponsive reward anticipation in the basal ganglia following severe institutional deprivation early in life. J. Cogn. Neurosci..

[bib0370] Mischel W., Ayduk O., Berman M.G., Casey B.J., Gotlib I.H., Jonides J., Kross E., Teslovich T., Wilson N.L., Zayas V., Shoda Y. (2011). “Willpower” over the life span: decomposing self-regulation. Soc. Cogn. Affect. Neurosci..

[bib0375] Moffitt T.E., Arseneault L., Belsky D., Dickson N., Hancox R.J., Harrington H., Houts R., Poulton R., Roberts B.W., Ross S., Sears M.R., Thomson W.M., Caspi A. (2011). A gradient of childhood self-control predicts health, wealth, and public safety. Proc. Natl. Acad. Sci. U. S. A..

[bib0380] Moilanen K.L., Shaw D.S., Dishion T.J., Gardner F., Wilson M. (2010). Predictors of longitudinal growth in inhibitory control in early childhood. Soc. Dev..

[bib0385] Morrisson F.J., Grammer J.K., Griffin J.A., McCardle P., Freund L. (2016). Conceptual clutter and measurement mayhem: proposals for cross-disciplinary integration in conceptualizing and measuring executive function. Executive Function in Preschool-Age Children: Integrating Measurement, Neurodevelopment, and Translational Research.

[bib0390] Newport E.L. (1990). Maturational constraints on language learning. Cogn. Sci..

[bib0395] Nigg J.T. (2017). Annual research review: on the relations among self-regulation, self-control, executive functioning, effortful control, cognitive control, impulsivity, risk-taking, and inhibition for developmental psychopathology. J. Child Psychol. Psychiatry Allied Discip..

[bib0400] Padmanabhan A., Geier C.F., Ordaz S.J., Teslovich T., Luna B. (2011). Developmental changes in brain function underlying the influence of reward processing on inhibitory control. Dev. Cogn. Neurosci..

[bib0405] Pandey A., Hale D., Das S., Goddings A.-L., Blakemore S.-J., Viner R.M. (2018). Effectiveness of universal self-regulation–based interventions in children and adolescents: a systematic review and meta-analysis. JAMA Pediatr..

[bib0410] Paus T. (2010). Growth of white matter in the adolescent brain: Myelin or axon?. Brain Cogn..

[bib0415] Penhune V., de Villers-Sidani E. (2014). Time for new thinking about sensitive periods. Front. Syst. Neurosci..

[bib0420] Pessoa L. (2009). How do emotion and motivation direct executive control?. Trends Cogn. Sci..

[bib0425] Pfeifer J.H., Allen N.B. (2012). Arrested development? Reconsidering dual-systems models of brain function in adolescence and disorders. Trends Cogn. Sci..

[bib0430] Piquero A.R., Jennings W.G., Farrington D.P., Diamond B., Gonzalez J.M.R. (2016). A meta-analysis update on the effectiveness of early self-control improvement programs to improve self-control and reduce delinquency. J. Exp. Criminol..

[bib0435] Posner M.I., Rothbart M.K. (2000). Developing mechanisms of self-regulation. Dev. Psychopathol..

[bib0440] Posner M.I., Rothbart M.K. (2007). Research on attention networks as a model for the integration of psychological science. Annu. Rev. Psychol..

[bib0445] Posner M.I., Rothbart M.K. (2018). Temperament and brain networks of attention. Philos. Trans. R. Soc. B Biol. Sci..

[bib0450] Posner M.I., Rothbart M.K., Sheese B.E., Voelker P. (2014). Developing attention: behavioral and brain mechanisms. Adv. Neurosci..

[bib0455] Posner M.I., Rothbart M.K., Voelker P. (2016). Developing brain networks of attention. Curr. Opin. Pediatr..

[bib0460] Rodriguez M.L., Aber J.L., Sethi A., Ayduk O., Shoda Y., Mischel W. (2005). A contextual approach to the development of self-regulatory competencies: the role of maternal unresponsivity and toddlers’ negative affect in stressful situations. Soc. Dev..

[bib0465] Rothbart M.K. (1981). Measurement of temperament in infancy. Child Dev..

[bib0470] Rothbart M.K., Bates J.E., Damon W., Eisenberg N. (1998). Temperament. Handbook of Child Psychology: Social, Emotional, and Personality Development.

[bib0475] Rothbart M.K., Posner M.I., Boylan A., Enss J. (1990). Regulatory mechanisms in infant development. The Development of Attention: Research and Theory.

[bib0480] Rothbart M.K., Ellis L.K., Rueda M.R., Posner M.I. (2003). Developing mechanisms of temperamental effortful control. J. Pers..

[bib0485] Rueda M.R., Posner M.I., Rothbart M.K. (2005). The development of executive attention: contributions to the emergence of self-regulation. Dev. Neuropsychol..

[bib0490] Samejima K., Doya K. (2007). Multiple representations of belief states and action values in corticobasal ganglia loops. Ann. N. Y. Acad. Sci..

[bib0495] Sameroff A. (2010). A unified theory of development: a dialectic integration of nature and nurture. Child Dev..

[bib0500] Sapienza J.K., Masten A.S. (2011). Understanding and promoting resilience in children and youth. Curr. Opin. Psychiatry.

[bib0505] Schoemaker K., Mulder H., Deković M., Matthys W. (2013). Executive functions in preschool children with externalizing behavior problems: a meta-analysis. J. Abnorm. Child Psychol..

[bib0510] Shannon K.E., Beauchaine T.P., Brenner S.L., Neuhaus E., Gatzke-Kopp L. (2007). Familial and temperamental predictors of resilience in children at risk for conduct disorder and depression. Dev. Psychopathol..

[bib0515] Sheridan M.A., Fox N.A., Zeanah C.H., McLaughlin K.A., Nelson C.A. (2012). Variation in neural development as a result of exposure to institutionalization early in childhood. Proc. Natl. Acad. Sci..

[bib0520] Somerville L.H., Casey B.J. (2010). Developmental neurobiology of cognitive control and motivational systems. Curr. Opin. Neurobiol..

[bib0525] Spear L.P. (2000). The adolescent brain and age-related behavioral manifestations. Neurosci. Biobehav. Rev..

[bib0530] Swingler M.M., Willoughby M.T., Calkins S.D. (2011). EEG power and coherence during preschoolers’ performance of an executive function battery. Dev. Psychobiol..

[bib0535] Swingler M.M., Perry N.B., Calkins S.D., Bell M.A. (2017). Maternal behavior predicts infant neurophysiological and behavioral attention processes in the first year. Dev. Psychol..

[bib0540] Teicher M.H., Anderson C.M., Ohashi K., Polcari A. (2014). Childhood maltreatment: altered network centrality of cingulate, precuneus, temporal pole and insula. Biol. Psychiatry.

[bib0545] van Duijvenvoorde A.C.K., Peters S., Braams B.R., Crone E.A. (2016). What motivates adolescents? Neural responses to rewards and their influence on adolescents’ risk taking, learning, and cognitive control. Neurosci. Biobehav. Rev..

[bib0550] Van Leijenhorst L., Moor B.G., Op de Macks Z.A., Rombouts S.A.R.B., Westenberg P.M., Crone E.A., Van Leijenhorst L., Crone E.A., Op de Macks Z.A. (2010). Adolescent risky decision-making: neurocognitive development of reward and control regions. Neuroimage.

[bib0555] Vazsonyi A.T., Pickering L.E., Junger M., Hessing D. (2001). An empirical test of a general theory of crime: a four-nation comparative study of self-control and the prediction of deviance. J. Res. Crime Delinq..

[bib0560] Vink M., Derks J.M., Hoogendam J.M., Hillegers M., Kahn R.S. (2014). Functional differences in emotion processing during adolescence and early adulthood. Neuroimage.

[bib0565] Vink M., Zandbelt B.B., Gladwin T., Hillegers M., Hoogendam J.M., van den Wildenberg W.P.M.M., Du Plessis S., Kahn R.S. (2014). Frontostriatal activity and connectivity increase during proactive inhibition across adolescence and early adulthood. Hum. Brain Mapp..

[bib0570] Vink M., de Leeuw M., Pouwels R., van den Munkhof H.E., Kahn R.S., Hillegers M. (2015). Diminishing striatal activation across adolescent development during reward anticipation in offspring of schizophrenia patients. Schizophr. Res..

[bib0575] Volkow N.D., Koob G.F., Croyle R.T., Bianchi D.W., Gordon J.A., Koroshetz W.J., Pérez-Stable E.J., Riley W.T., Bloch M.H., Conway K., Deeds B.G., Dowling G.J., Grant S., Howlett K.D., Matochik J.A., Morgan G.D., Murray M.M., Noronha A., Spong C.Y., Wargo E.M., Warren K.R., Weiss S.R.B., Deesds B.G., Dowling G.J., Grant S., Howlett K.D., Matochik J.A., Morgan G.D., Murray M.M., Noronha A., Spong C.Y., Wargo E.M., Warren K.R., Weiss S.R.B. (2018). The conception of the ABCD study: from substance use to a broad NIH collaboration. Dev. Cogn. Neurosci..

[bib0580] Whedon M., Perry N.B., Calkins S.D., Bell M.A. (2016). Changes in frontal EEG coherence across infancy predict cognitive abilities at age 3: the mediating role of attentional control. Dev. Psychol..

[bib0585] Wierenga L.M., Langen M., Oranje B., Durston S. (2014). Unique developmental trajectories of cortical thickness and surface area. Neuroimage.

[bib0590] Zandbelt B.B.B., Vink M. (2010). On the role of the striatum in response inhibition. PLoS One.

[bib0595] Zhou Q., Chen S.H., Main A. (2012). Commonalities and differences in the research on children’s effortful control and executive function: a call for an integrated model of self-regulation. Child Dev. Perspect..

